# Patient Perspectives on Medical Trainee Involvement in the Anesthesia-Related Procedural Skill Attainment: A Cross-Sectional Study

**DOI:** 10.7759/cureus.94730

**Published:** 2025-10-16

**Authors:** Michael V Balce, Adam J Cruz, Patricia Rusli, Karen L Childers, Roy G Soto

**Affiliations:** 1 Anesthesiology, Corewell Health William Beaumont University Hospital, Royal Oak, USA; 2 Anesthesiology, Oakland University William Beaumont School of Medicine, Rochester, USA; 3 Anesthesiology, University of Michigan Hospital, Ann Arbor, USA; 4 Anesthesiology, The Ohio State University Wexner Medical Center, Columbus, USA; 5 Biostatistics, Corewell Health William Beaumont University Hospital, Royal Oak, USA

**Keywords:** anesthesia, ethics, informed consent, medical education, shared decision making, skill attainment

## Abstract

Introduction

This qualitative study examines patient preferences in trainee involvement with various anesthesia-related procedures (endotracheal intubations, fiberoptic intubations, epidurals, arterial lines, and central lines) after they are educated on the details of these procedures. The goal of the study is to observe patients’ wishes regarding trainee involvement in care. This study predicted that most participants would express reluctance toward trainee procedural involvement and would prefer trainees to have at least 10 repetitions of a procedure before performing it on them.

Methods

The study was conducted between March and June of 2022 at Corewell Health William Beaumont University Hospital in Royal Oak, USA. As part of the study, 100 participants partook in a questionnaire that assessed their knowledge of training differences between medical students, residents, fellows, and attending physicians. The participants were educated on features of the aforementioned anesthesia-related procedures (endotracheal intubations, fiberoptic intubations, epidurals, arterial lines, and central lines), and were then administered a questionnaire that inquired about their preferences regarding trainee participation in these treatments.

Results

Of the participants surveyed (N = 100), 87% agreed to have trainees gain experience from their care. Before trainee procedural involvement, many participants preferred that trainees have completed 5 to 10 repetitions of a procedure. Regarding epidural procedures, 30% of participants stated that they never wanted trainees involved. When asked about levels of training, 47% of participants understood the difference between a resident and a fellow, and 51% understood the difference between fellows and attending physicians. Additionally, 76% of participants expressed a desire for the distinctions between medical students, residents, fellows, and attendings to be explained to them when these individuals are involved in their care.

Conclusion

Participants did not express reluctance toward trainee procedural involvement and were accepting when trainees had fewer than 10 repetitions of a procedure before performing it on them. When asked about their prior knowledge of the definitions of trainees, participants were unlikely to know the difference in the training level of a fellow compared to a resident or an attending. Therefore, explaining the title and training history of those involved in a procedure may help improve clarity for patients regarding who is involved in their care. The implications of these findings may open the door to studies regarding patient opinions on trainee involvement in treatments from medical specialties other than anesthesiology.

## Introduction

“See one, do one, teach one” has been a commonplace refrain since the late 1800s, when William Halsted used it to describe his method of surgical resident education [1]. Most physicians and medical students are familiar with this saying, but few patients are likely to be aware of what it means for their care. Patients may know they are being treated at teaching hospitals, but they may not understand which members of their treatment team are trainees, how much training each team member has received, or whether trainees will be directly providing interventions [2]. Informed consent is about agreement to a proposed procedure after presentation of risks, benefits, and alternatives by a party proposing to perform the procedure [3-5]. In particular, surgeons and anesthesiologists provide patients with information about proposed perioperative interventions. In teaching hospitals, medical students, residents, and fellows participate directly in patient care under the direction of senior physicians. Informed consent at these institutions should, therefore, include a disclosure to patients that trainees will be participating in their care [6]. Such disclosures are commonly made; however, patients are less frequently informed about trainees’ level of skill or experience during the consent process for a procedure [7,8]. If patients do not understand the expertise and experience of the trainees who are participating in interventions, this may undermine patients' ability to provide meaningful informed consent [9].

This study aimed to evaluate patients’ understanding of the medical training hierarchy and to assess patients’ comfort with anesthesiology trainees performing common anesthesia-related procedures. The authors of this project expected most patients to express reluctance toward relatively inexperienced trainees performing procedures, based on perceptions at the hospital where this study took place.

## Materials and methods

We designed a cross-sectional study utilizing a patient questionnaire from March to June of 2022 (Appendix A). The survey contained questions regarding how comfortable patients felt with trainees being involved in performing endotracheal intubation, elective asleep fiberoptic intubation, epidural placement, radial artery cannulation, and central venous cannulation. It was explained to the participants that trainee involvement in these procedures almost always included an attending anesthesiologist nearby, providing direct supervision. Each procedure included a written description in lay terms, describing key aspects of the procedure, in addition to the relative rarity of the most common complications. Additionally, participants took a test on the survey to ascertain their understanding of the differences between medical students, residents, fellows, and attending physicians.

The questionnaire was distributed in the recovery room (Phase 2 area, after receiving post-operation instructions and immediately prior to discharge) at Corewell Health William Beaumont University Hospital in Royal Oak, USA (a tertiary care, teaching hospital). Potential participants in the study were asked whether they would complete a questionnaire about medical trainees performing hypothetical anesthesiology-related procedures on them. To minimize bias, all eligible patients in the recovery room were approached for participation. Excluded populations included decisionally impaired adults (patients with capacity limitations due to residual sedation, as well as other causes of diminished capacity) and individuals less than 18 years of age (Appendix B). All participants were assessed to ensure full capacity via assessment of understanding, appreciation, reasoning, and communication regarding participation in the research study. Additionally, delirium was assessed via the CAM-IMC (Confusion Assessment Method for Intermediate Care Unit) assessment prior to discharge and right before being administered the survey [10]. The Beaumont Health Institutional Review Board issued approval for this study (approval no. 2021-350).

A convenience sample of 106 patients was randomly approached by two of the authors, and 100 patients completed the survey. Six patients either declined to participate or were not eligible (two refused, and four were ineligible). Data collection was completed once 100 surveys were collected. Participants in the study were asked questions intended to collect demographic data, assess their knowledge of the roles of medical students, residents, and fellows, and evaluate their comfort level with trainee experience and procedures.

For survey questions, the percentage of respondents who chose each answer was calculated and displayed graphically. R version 3.5.1 (R Foundation for Statistical Computing, Vienna, Austria) was used for analyses and figures.

## Results

**Figure 1:**
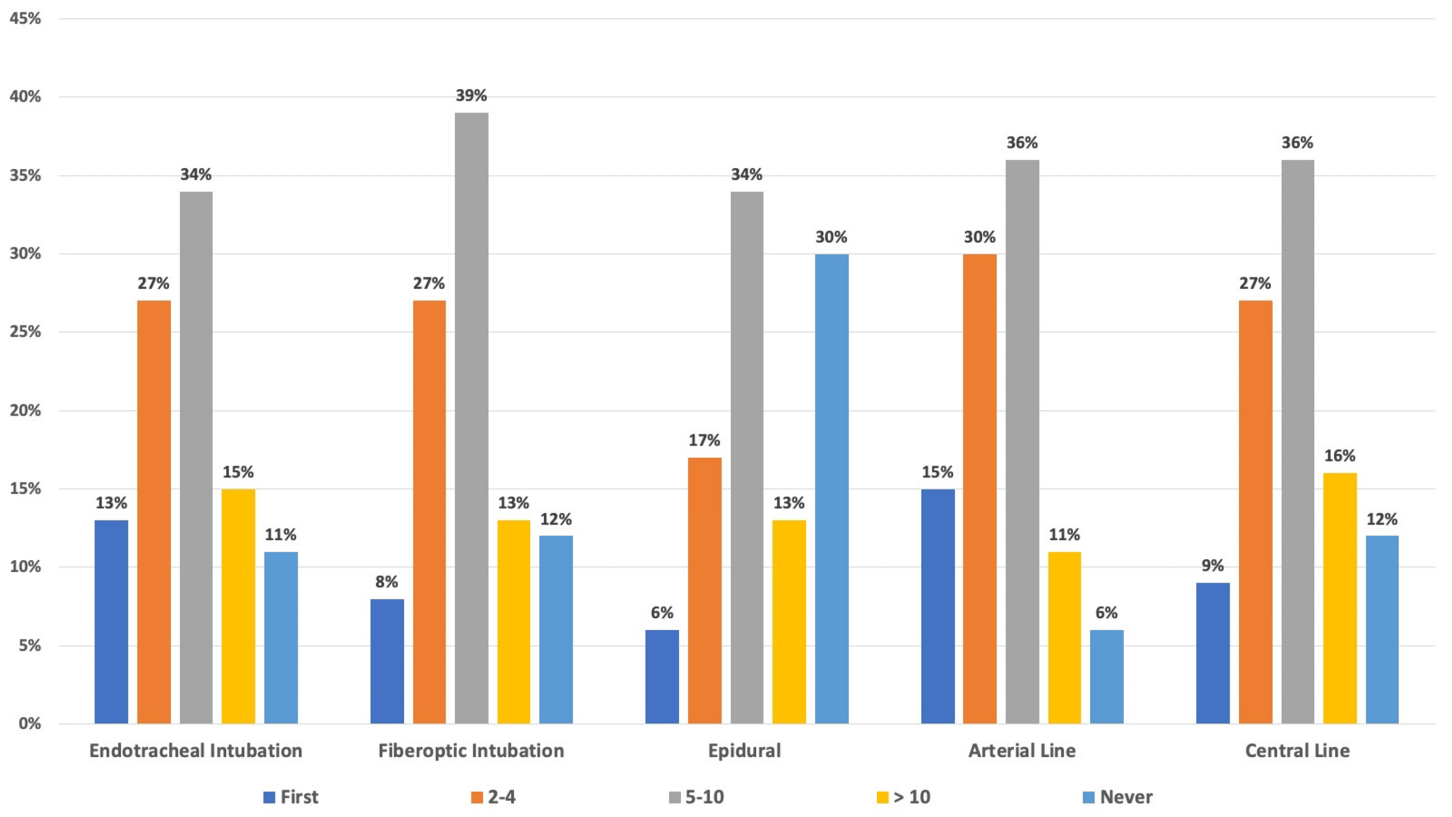
Number of Procedures Participants Wanted Trainees to Undergo Before Having the Procedures Performed on Them Each column is represented by the percentage (%) of participants’ (N = 100) answers regarding the number of repetitions (First Attempt, 2-4, 5-10, Greater Than 10, Never Want Learners to Attempt) of a procedure (Endotracheal Intubation, Fiberoptic Intubation, Epidural, Arterial Line, Central Line) within the survey.

A total of 106 patients were asked to participate in the study, and 100 agreed (94.3% participation rate). Responses regarding trainee experience prior to allowing a procedure to be performed were grouped into “first, 2-4, 5-10, greater than 10, or never,” as seen in Figure 1. While the majority of responders (N = 100) wanted trainees to have performed at least 5-10 repetitions of each of the procedures before those procedures were performed on them, the only procedure that over 15% of participants never wanted trainees to be involved with was the epidural (30% never).

**Figure 2:**
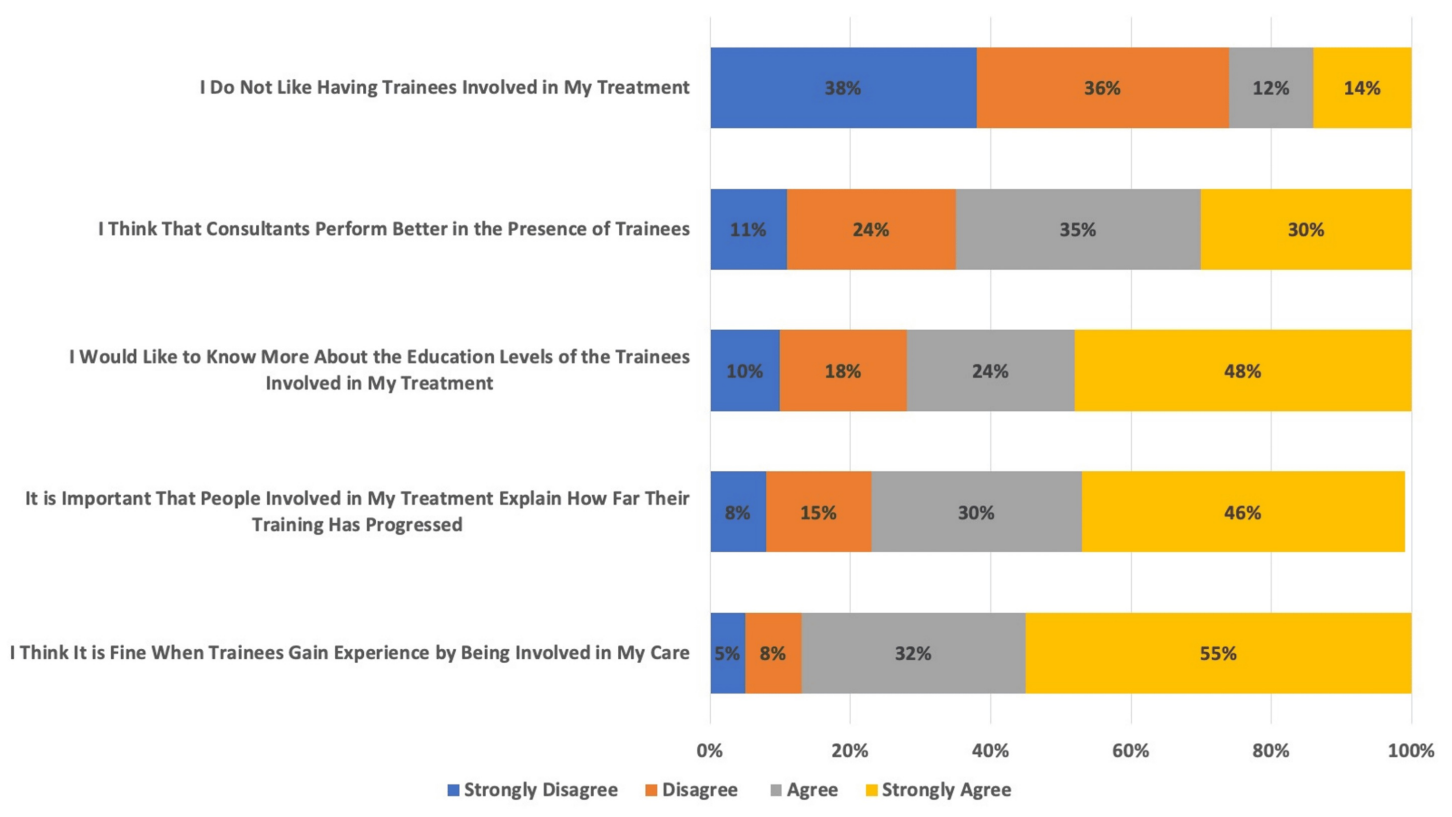
Participant Opinions on Trainee Involvement Each row is represented by the percentage (%) of participants’ (N = 100) answers - Strongly Disagree, Disagree, Agree, Strongly Agree - to each of these statements within the survey.

In Figure 2, results for the desire to have trainees involved in care were categorized by procedure (endotracheal intubation, fiberoptic intubation, epidural, arterial line, and central line). Within each category, participants (N = 100) answered questions about each procedure in which they “strongly agreed, agreed, disagreed, or strongly disagreed.” Regarding overall trainee involvement, only 13% of the participants did not want trainees to be involved with their care (87% agreed to trainees being engaged with care). The 13% who preferred no trainees be involved had documented requests to exclude trainees from surgical or anesthetic procedures prior to being asked in the survey. Of note, the response “neither agree nor disagree” was omitted from this graphic.

**Figure 3:**
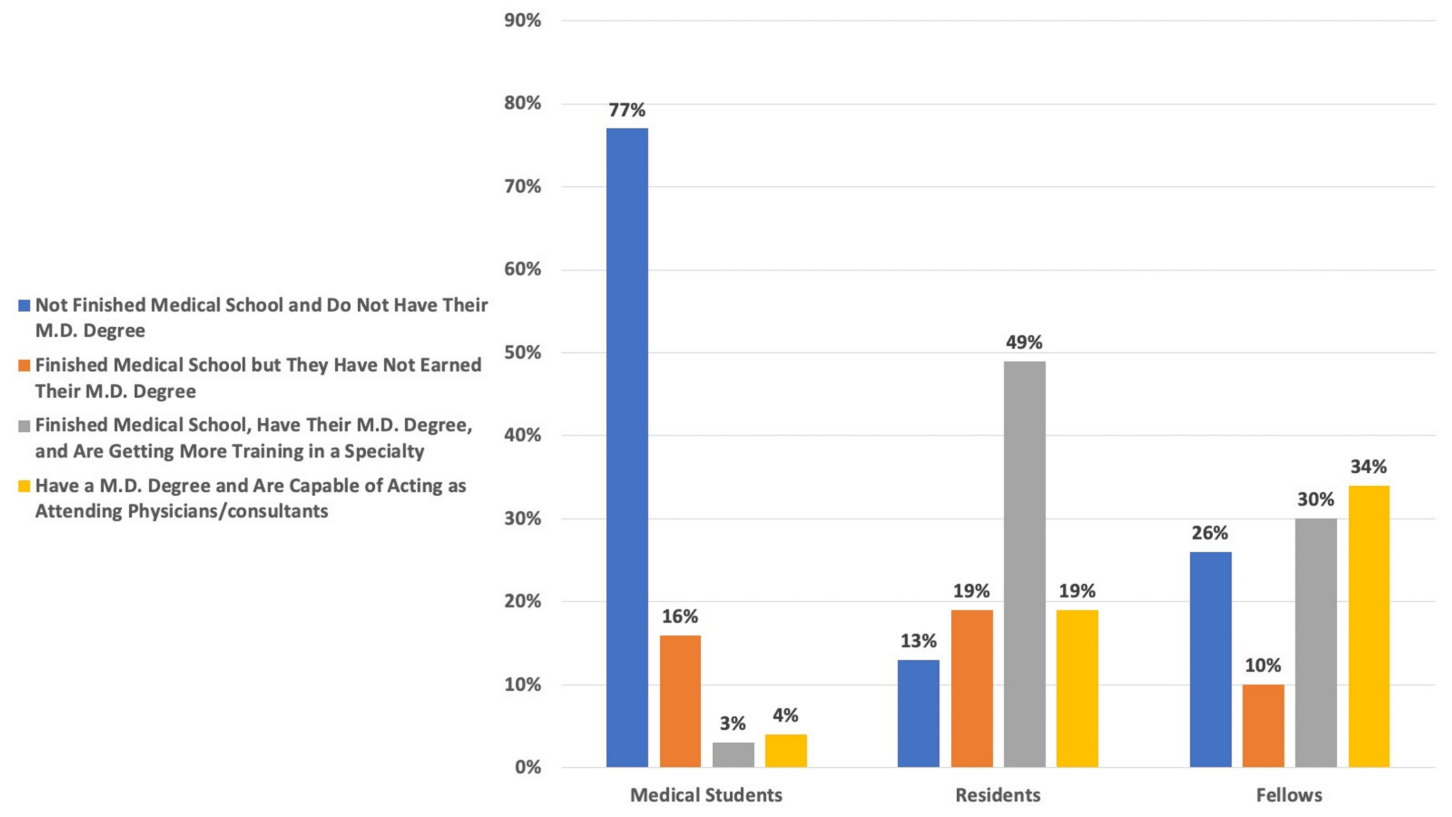
Participant Knowledge of the Training Criteria of Medical Students, Residents, and Fellows Each column is represented by the percentage (%) of participants' (N = 100) answers regarding the difference in level of education between medical trainees (Medical Student, Resident, Fellow).

The results from the participants’ (N = 100) perceptions of the educational levels of the trainees are listed in Figure 3. While most participants understood that medical students have not finished medical school and do not have an MD/DO degree (77%), participants were less likely to understand the training level of fellows.

**Figure 4:**
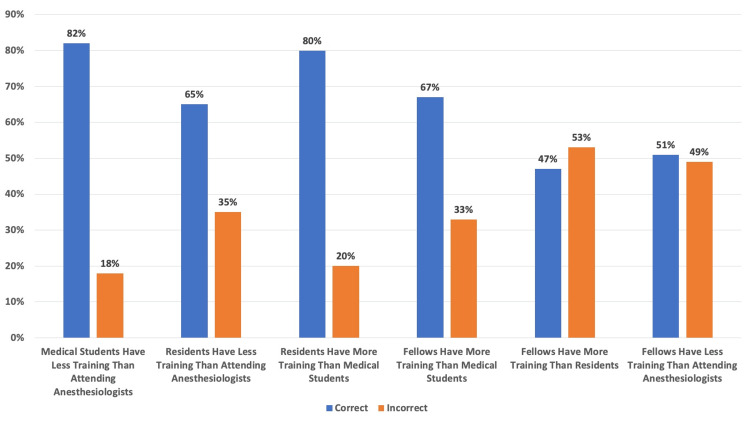
Correct Versus Incorrect Participant Response Rate on Who Received More Training Each column is represented by the percentage (%) of participants' (N = 100) correct versus incorrect answers when comparing the level of training between medical students, residents, fellows, and attending anesthesiologists.

The participants’ (N = 100) correct and incorrect rates in comparing the levels of training between medical students, residents, fellows, and anesthesiologists are shown in Figure 4. The correct versus incorrect rates of training level identification were similar between fellows and residents (47% correctly identified the difference) and fellows and attending anesthesiologists (51% correctly identified the difference). No associations were found between participants’ demographics and their response rates.

## Discussion

Informed consent, an essential principle of medical ethics in healthcare today, enshrines patients’ rights to make educated decisions about their medical care based on their wants and desires [11]. Through shared decision-making with a consulting physician, informed consent highlights the principle of autonomy, since a patient’s values and opinions guide his or her treatment decisions [12]. In teaching hospitals, patients are generally willing to have medical students, residents, and fellows practice and learn through their care [13]. However, patients often lack a clear and concise understanding of the trainee’s educational background and familiarity with a procedure [14,15]. The goal of our study was to obtain patient viewpoints on medical trainee participation in anesthesia-related procedural skill attainment.

We anticipated that most participants would be too reluctant to have relatively inexperienced trainees perform their procedures. However, we found that most participants agreed to allow trainees to learn clinical skills through their treatment, regardless of trainee experience. While there seems to be increased confidence in having trainees involved in patients’ care with more experience, many participants were willing to be the first, or close to the first, patient on whom a trainee performs a procedure. Additionally, most participants in the study preferred trainees to undergo 5-10 repetitions of the procedures (endotracheal intubation, elective asleep fiberoptic intubation, epidural placement, radial artery cannulation, central venous cannulation) before having each procedure performed on them as well. 

Conversely, patients felt strongly about trainees not being involved with epidural placement, as opposed to the other procedures in the questionnaire (endotracheal intubation, fiberoptic intubation, arterial line, central line), which could potentially be due to the perceived invasiveness (or lay understanding) of the procedure [16]. When patients were asked to differentiate between a medical student, resident, fellow, and attending anesthesiologist, most participants were unable to identify the level of training that a fellow has compared to that of a resident or attending anesthesiologist.

The four pillars of clinical ethics are autonomy, justice, beneficence, and nonmaleficence [17]. Autonomy, in particular, is highlighted in the shared decision-making process because it acknowledges that patients have the right to make decisions regarding whether or not to undergo procedures the physician offers them, regardless of whether the physician agrees with the decision [18]. Informed consent, in the current state of healthcare, is crucial when providing medical treatments so that the relationship between physician and patient prioritizes trust, respect, and transparency [19,20]. Thoughtful conversations when obtaining informed consent ensure that a patient’s wishes are at the center of medical care, that there is ongoing communication and trust between provider and patient, and that a patient understands all the pertinent details regarding his or her treatment decisions [21].

As mentioned, components of informed consent include treatment details, risks and benefits of the treatment, risks and benefits of treatment alternatives, and a patient’s understanding of the treatment [22]. While the current requirements for informed consent are comprehensive with regard to a patient’s willingness to undergo a procedure, consent discussions often do not highlight the relevant clinical background and experience of who will be performing said treatment [23].

With this in mind, a patient could experience diminished feelings of liberty when making decisions about whether or not to undergo an offered procedure without knowing the proficiency of the practitioner carrying out the procedure [24]. Even if one’s abilities are discussed with a patient, our study suggests that there are times when certain titles - particularly fellows - are indistinguishable from other levels of trainees. This allows for the potential for patients to consent to procedures without due understanding of who will be performing the said procedure. 

Solutions to the potential lack of self-determination patients may feel when deciding whether or not to undergo a procedure, due to questioning trainee proficiency, should focus on increased transparency during conversations with the provider. For example, the American College of Obstetricians and Gynecologists has taken a leadership role in addressing the current limitations in consent discussions as they pertain to trainees. In 2025, they issued a committee opinion on the indications for pelvic examinations by trainees under anesthesia [25]. They clearly affirmed that the status of personnel performing pelvic exams under anesthesia should be disclosed prior to a surgical procedure. This type of transparency should increase patient autonomy, positively supplement patients’ desire to have trainee involvement with their treatment, and build trust during shared decision-making between the patient and a medical professional.

Limitations

As with all studies, ours comes with limitations. These limitations include our hospital setting. Since patients are specifically coming to a single university hospital (rather than multiple centers or ones without trainees), one could assume that patients would be more inclined to agree to trainee involvement. Results might be different at a community or private hospital. Although trainees frequently rotate to these centers, patients may be less aware of, and less willing to participate in, education than those at named teaching hospitals. Additionally, the patients selected to be participants in the study were limited to those who underwent urologic, gynecologic, neurologic, or otolaryngologic procedures due to recovery room assignment. Results from different types of surgical procedures involving specialties other than those included in our study could change patient perspectives on anesthesia trainee involvement. Another interesting limitation we uncovered comes from patients’ desire not to have epidurals placed by trainees. Our survey did not explicitly mention labor epidurals, and it would be interesting to learn if acute pain and urgency would make patients more or less willing to allow trainee care. Furthermore, patients were solely given the survey after their procedures rather than before. A participant’s decisions in the survey may have differed if it had been given at a different point in his or her surgical timeline. However, when the researchers attempted to administer the survey before the participants’ procedures, it caused unnecessary distress for potential participants. It was therefore decided that the survey would be administered post-procedure. Pre-procedure, patients felt as though the survey was a stressful, time-consuming event during the perioperative experience - especially when potential participants expressed they would rather spend time with family and speak with providers pre-surgery instead of being asked hypothetical questions in a research study. Some confounding factors that may have influenced the results in our study include prior experiences with anesthesia, patient education level, and exposure to teaching hospitals in previous care. Finally, we did not address consent by proxy. When families make medical decisions for patients (e.g., in an ICU setting), they may or may not be more willing to allow trainee care.

## Conclusions

In conclusion, we found that patients were willing to allow trainees to be involved in their care, although their understanding of the level of trainee - particularly that of a fellow compared to residents or attending anesthesiologists - was not as robust as it could be. Some procedures, particularly epidurals, made patients less comfortable with trainee involvement. However, most patients did understand that trainee care was appropriate, at least at our institution. We feel strongly that increased transparency during patient consent is always a good approach.

## Figures and Tables

**Figure 5:**
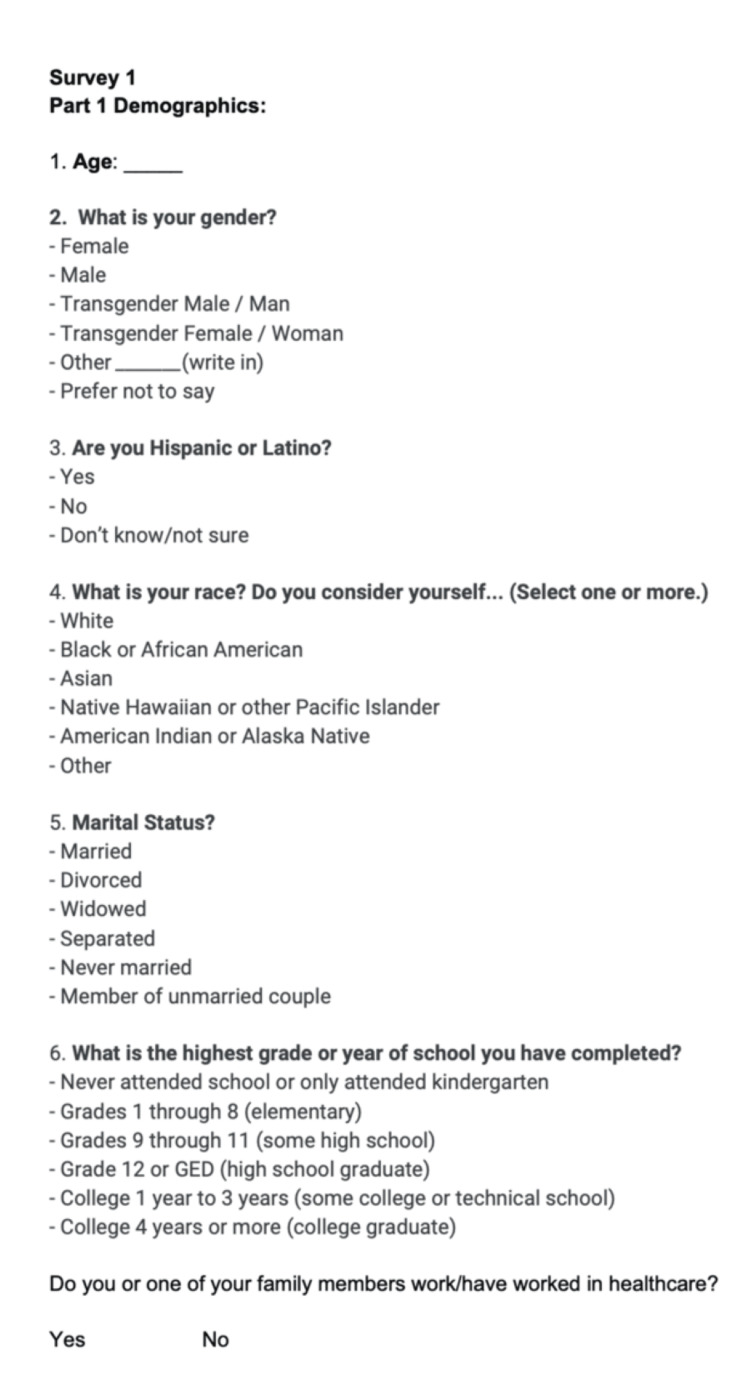
Survey Page 1

**Figure 6:**
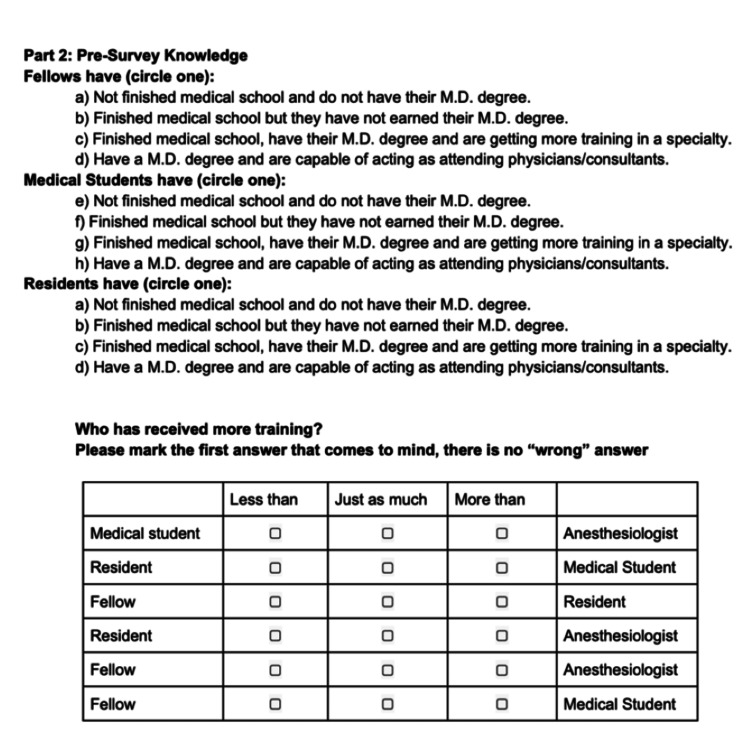
Survey Page 2

**Figure 7:**
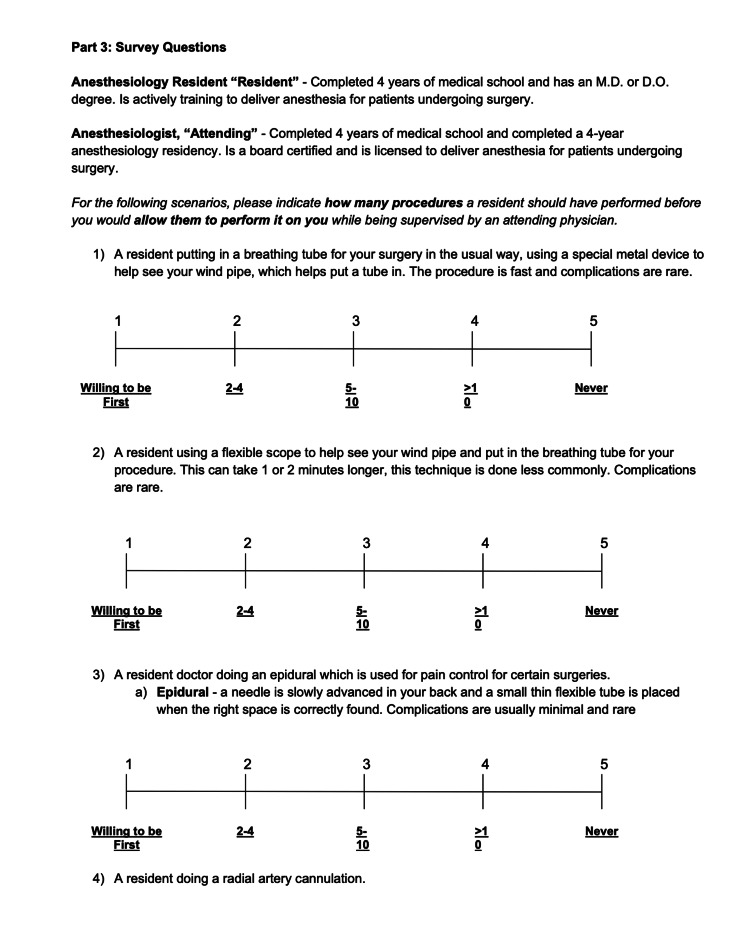
Survey Page 3

**Figure 8:**
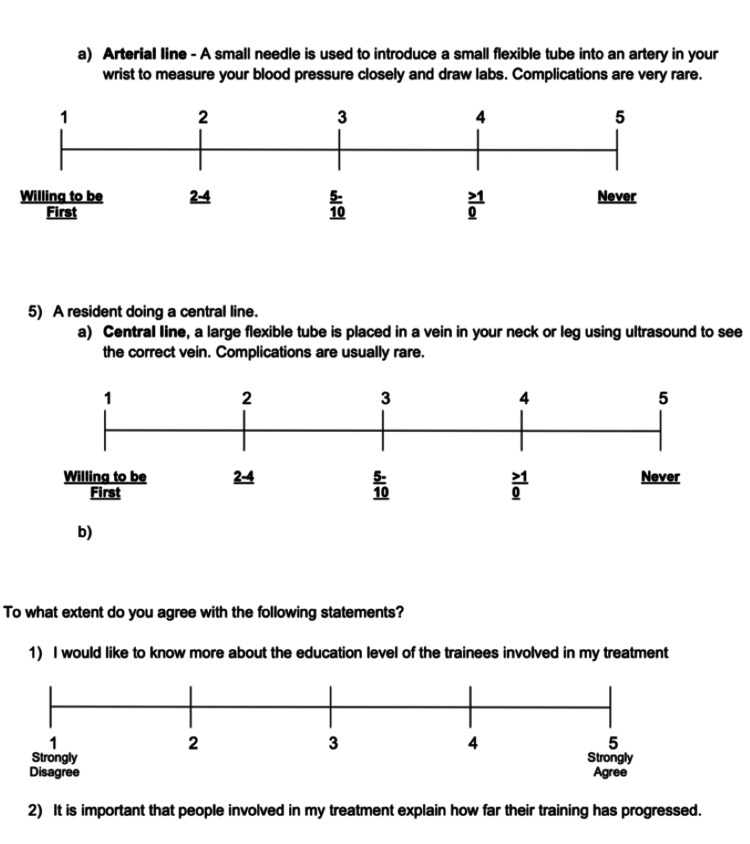
Survey Page 4

**Figure 9:**
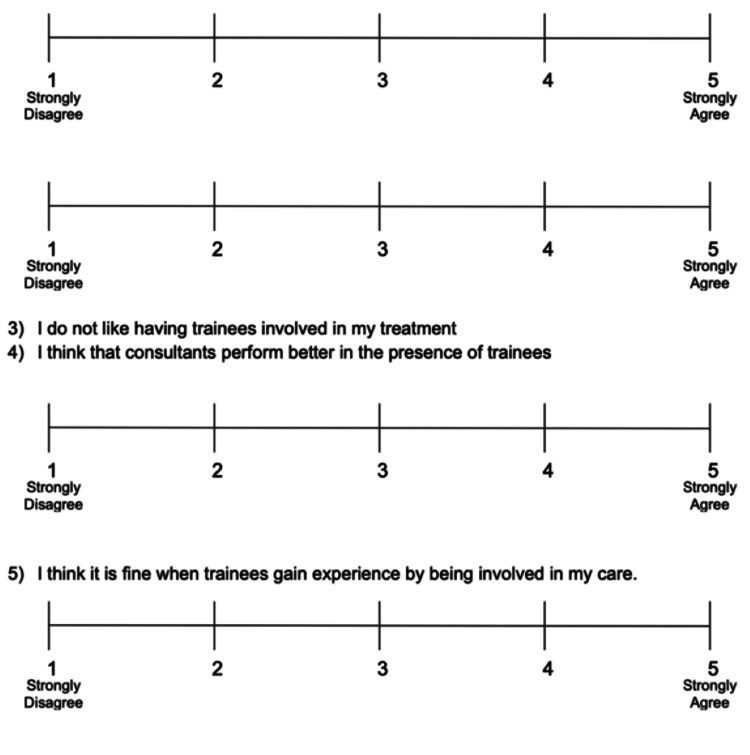
Survey Page 5

**Figure 10:**
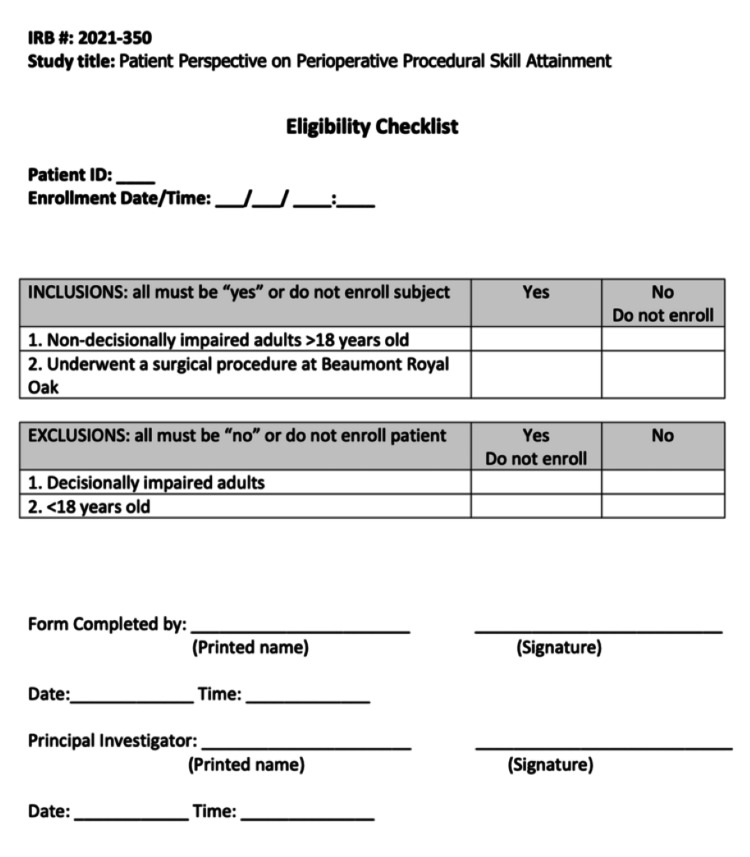
Participant Eligibility Checklist

## Appendices

Appendix A

Appendix B
